# Pyridine nucleotide regulation of hepatic endoplasmic reticulum calcium uptake

**DOI:** 10.14814/phy2.14151

**Published:** 2019-06-20

**Authors:** Xudong Wang, Gail Mick, Kenneth McCormick

**Affiliations:** ^1^ Department of Pediatrics University of Alabama at Birmingham Birmingham Alabama

**Keywords:** pyridine nucleotides, calcium uptake, microsomes

## Abstract

Pyridine nucleotides serve an array of intracellular metabolic functions such as, to name a few, shuttling electrons in enzymatic reactions, safeguarding the redox state against reactive oxygen species, cytochrome P450 (CYP) enzyme detoxification pathways and, relevant to this study, the regulation of ion fluxes. In particular, the maintenance of a steep calcium gradient between the cytosol and endoplasmic reticulum (ER), without which apoptosis ensues, is achieved by an elaborate combination of energy–requiring ER membrane pumps and efflux channels. In liver microsomes, net calcium uptake was inhibited by physiological concentrations of NADP. In the presence of 1 mmol/L NADP, calcium uptake was attenuated by nearly 80%, additionally, this inhibitory effect was blunted by concomitant addition of NADPH. No other nicotinamide containing compounds ‐save a slight inhibition by NAADP‐hindered calcium uptake; thus, only oxidized pyridine nucleotides, or related compounds with a phosphate moiety, had an imposing effect. Moreover, the NADP inhibition was evident even after selectively blocking ER calcium efflux channels. Given the fundamental role of endoplasmic calcium homeostasis, it is plausible that changes in cytosolic NADP concentration, for example, during anabolic processes, could regulate net ER calcium uptake.

## Introduction

The coupled redox pyridine nucleotides, NAD^+^/NADH and their phosphorylated congeners NADP^+^/NADPH, oversee manifold biochemical functions in eukaryotic cells. When reduced, pyridine nucleotides serve as universal electron donors in enzymatic reactions. These ubiquitous cofactors can shuttle two electrons in oxidoreductase reactions, and are fundamental in the anabolic reductive synthesis of fatty acids, phospholipids, steroids, and some amino acids. Other eminent roles for these cofactors include defending against reactive oxidative species, cytochrome P450 enzymatic detoxification, and the modulation of signal transduction.

Insofar as the maintenance of intracellular calcium gradients in several organelles is a requisite for cell viability, it is plausible that pyridine nucleotides also regulate calcium fluxes (Zima et al. 2004; Vangheluwe et al. 2005; Kilfoil et al. 2013; Raffaello et al. 2016). Relative to the cytosol, high ER calcium concentrations are indispensable for cell differentiation and proliferation, the synthesis and chaperone‐assisted folding of proteins, activity of oxidoreductases, and possibly gene transcription. Dysregulation of intracellular calcium homeostasis begets ER stress and apoptosis.

In the liver, the endoplasmic reticulum (ER) is the major calcium reservoir, and the concentration therein is regulated by the integration of antithetical fluxes across the membrane. P_2_‐ATPase ion transporters uphold the steep calcium concentrations in the ER. Influx from the cytosol to the ER occurs via a family of P_2_‐ATPase ion transporters, and efflux through discrete ion channels. Considering the ER luminal free calcium concentration of ~400 μmol/L, which is a 1000‐fold over that found in the cytosol, this steep gradient consumes considerable ATP energy. A family of membrane anchored sarco/endoplasmic calcium ATPase (SERCA) pumps sustain the gradient. Serca2b is the isoform present in hepatic microsomes (Anger et al. 1994; Vangheluwe et al. 2005; Vandecaetsbeek et al. 2011).

In addition to a direct pyridine nucleotide effect on calcium channels, their metabolic derivatives may have similar actions. For instance, two derivatives of the nicotinamide NADP, namely, NAADP and ADPR, stimulate calcium efflux from cell organelles, including liver ER (Galione and Ruas 2005; Mandi et al. 2006). Consequently, insofar as NADP inhibits net ER calcium uptake, it is conceivable that the effect could be a result of increased calcium egress, not ingress, resulting from the production of the two aforementioned metabolites.

In vitro experimental approaches must approximate extant eukaryotic pyridine nucleotide concentrations, and their ratios, in disparate tissue organelles. Contingent on the subcellular pool (for example, cytosol, mitochondria, or ER), the phosphorylated or nonphosphorylated pyridine redox ratios can differ logarithmically (>10^4^) (Somogyi et al. 2016). And, accordingly, the ratios therein dictate the subcellular metabolic processes in each domain (Hosios and van der Heiden 2018). To illustrate, cytosolic glycolysis versus ER protein folding and ER cortisol production transpire under considerable differences in pyridine redox ratios. Estimates of the hepatic concentrations, and ratios, of the nucleotides within the intracellular compartments vary – discussed at length in (Goodman et al. 2018)‐ but reportedly the phosphorylated nucleotide NADP^+^/ NADPH ratio(.001‐.01) is reduced in both the ER and the cytosol. Paradoxically, this reduced ER ratio somehow coexists with an otherwise luminal oxidized milieu (Margittai et al. 2015). Antithetically, the nonphosphorylated NAD^+^/NADH ratio in the cytosol is purportedly well above 500, favoring carbon flux through glycolysis.

Bearing in mind these aforesaid pyridine nucleotide concentrations, their effect on net calcium uptake in liver microsomes was assessed under disparate assay conditions in which either efflux channels were blocked, the redox or pH milieu was altered, or upon adjusting the free calcium concentration.

## Materials and Methods

^45^CaCl_2_ was purchased from American Radiolabeled Chemicals. D9 (thioredoxin reductase inhibitor) was from Bio‐Techne corporation. Other compounds and general chemicals were from Sigma–Aldrich.

### Preparation of rat liver microsomes

Microsomes were isolated from the liver of Sprague Dawley rats (150–200 g body weight) as previously described (McCormick et al. 2006; Wang et al. 2011). All animal procedures were undertaken with approval and oversight of the Institutional Animal Care and Use Committee (IACUC) at the University of Alabama Birmingham. Tissue samples were homogenized in an ice‐bath with 4 vol. of 0.25 mol/L sucrose and 50 mmol/L Tris/HCl, pH 7.3. The homogenate was centrifuged for 10 min at 1000*g*. The supernatant portion was removed and centrifuged for 20 min at 10,000*g*. and, thereafter, centrifuged for 60 min at 100,000*g*. The resulting pellet was washed twice with the same buffer. The microsomes were resuspended at a protein concentration of 15–20 mg/mL (measured using a Bio‐Rad protein assay).

### Calcium uptake

Calcium uptake was measured in the following medium: 100 mmol/L KCl, 10 mmol/L MOPS buffer, pH 7.2, 5 mmol/L sodium azide, 5 mmol/L MgC1_2_, 1 mmol/L ATP, 5 mmol/L creatine phosphate, 5 units/mL creatine phosphokinase, 5 mmol/L ammonium oxalate and 100 μmol/L CaCl_2_ (100 μmol/L free calcium) and 0.1 μCi/mL of ^45^CaC1_2_ in a total volume of 0.4 mL. The assay was conducted at 37°C and initiated with the addition of the microsomes to a concentration of 0.1 mg of protein/mL. The reactions were terminated after 30 min by filtering through 0.45 μm membrane filters (Millipore Corp.) and the filters were washed with 0.25 mol/L sucrose (2 mL). The filters were dried and ^45^Ca^2+^ was determined using liquid scintillation counter (PerkinElmer) (Moore et al. 1975). Under these standard conditions, namely, 0.1 mg/mL protein and 30 min incubation, the control rate was 5.42 ± 0.19 (SEM) nmol/min/mg protein, similar to those previously reported in liver microsomes. Of note, if ATP is omitted there was no microsomal calcium uptake. The effect of various redox modifiers (at 1 mmol/L) on calcium uptake were tested, including: ascorbate (Vergauwen et al. 2015; Chen and Chang 2018), GSH and GSSG (Jessop and Bulleid 2004; Saaranen et al. 2010; Foyer and Noctor 2011), dehydroascorbate (DHA) (Csala et al. 1999; Saaranen et al. 2010; Foyer and Noctor 2011), H_2_O_2_ (Xu et al. 1997; Csala et al. 1999), and dithiothreitol (DTT) (Ushioda et al. 2016).

### Ca^2+^ATPase activity

Total ATPase activity (both Mg^2+^ and Ca^2+^ in the assay) of liver microsomes was measured with a direct colorimetric assay (Chan et al. 1986). The reaction was initiated by adding 0.1 mg/mL liver microsomes to the assay buffer containing 0.2 mmol/L EGTA, 1 mmol/L Mg^2+^ATP, 5 mmol/L NaN_3_, 2.5 μmol/L ruthenium red, and CaCl_2_ to yield 20 μmol/L free calcium in a buffer of 100 mmol/L KCl and 50 mmol/L imidazole, pH 6.8. In case of any mitochondrial contamination of the microsomal preparation, sodium azide was added to quell calcium uptake by this organelle. After 15 min of incubation at 37°C, 50 μL of this reaction mixture was added to 200 μL assay mix (malachite green), the absorbance of the color complex was measured at 630 nm with a microplate reader (Bio‐Rad). Pi quantity was determined from a standard curve prepared with known amounts of KH_2_PO_4_; the reaction was linear to at least 12.5 nmols P_i_. The Mg^2+^‐ATPase component was determined by subtracting the control value (no calcium added) from the total ATPase activity.

### Calcium release

Microsomes were washed once with 6% PEG and diluted to 15 mg/mL with KCl‐MOPS buffer containing 100 mmol/L KCl, 20 mmol/L MOPS, 20 mmol/L NaCl, 1 mmol/L MgCl_2_, pH 7.2, preloaded with 1 mmol/L CaCl_2_ plus 40 μCi/mL ^45^CaCl_2_ for 1 h at room temperature. The microsomal suspensions were then diluted 1:100 with the KCl‐MOPS buffer to activate the efflux. At different time points, 0.4 mL of suspensions was applied on the 0.45 μm membrane filters and then washed with 3 mL ice‐cold buffer containing 0.25 mol/L sucrose, 60 mmol/L Tris‐HCl, 1 mmol/L LaCl_3_, pH 7.2. The filters were dried and ^45^Ca^2+^ was determined using liquid scintillation counter (Scherer and Deamer 1986; Giunti et al. 2007).

### Statistical analysis

Data are presented as mean ± SEM (*n*). The significance of differences between groups was determined using Student's *t* test and analysis of variance. A *P* < 0.05 value was the cutoff level to reject the null hypothesis. All statistical analyses were performed with GraphPad Prism software (San Diego, CA, USA). Mean control values were nominally assigned a value of 100%.

## Results

**Figure 1:**
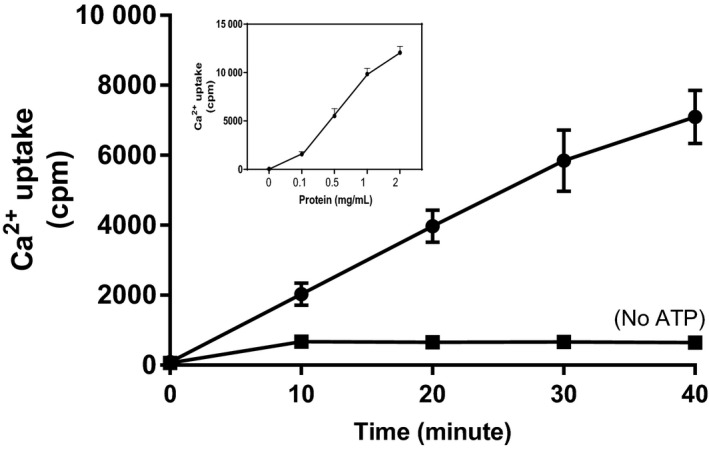
Time and protein relationships of liver microsomal calcium uptake. Microsomal protein (0.5 mg/ml) was used for the time graph, and 30 min incubation time for the protein graph (insert). Results are expressed as mean ± SEM.

Figure 1 depicts the time/microsomal protein relationships with calcium uptake in rat liver microsomes. At 0.5 mg microsomal protein, the uptake was linear for 30 min, and up to 40 min at 0.1 mg protein (data not shown for the 0.1 mg protein). Basal calcium uptake rate was 5.42 ± 0.19 nmol/mg protein/ min, similar to previous reports (Brattin et al. 1982; Erickson et al. 1987; Moore et al. 1975; Thastrup et al. 1990; Mandi et al. 2006). In the absence of ATP, the uptake was minimal.

**Figure 2:**
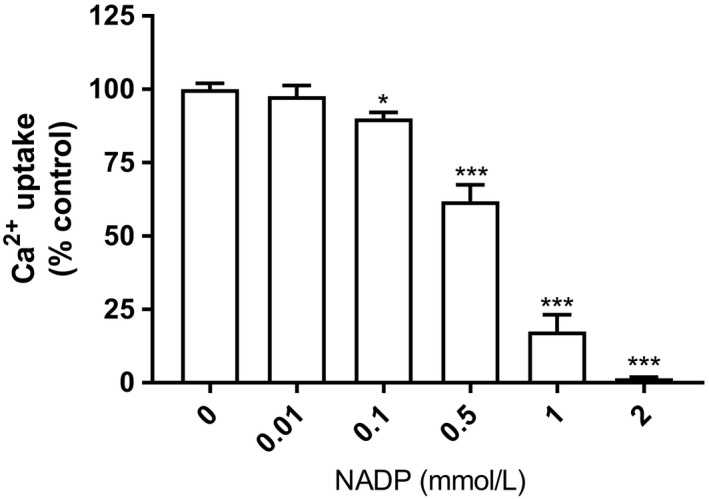
Effect of NADP and NAD on liver microsomal Ca^2+^ uptake. Isolated rat liver microsomes were incubated with the indicated concentrations of NADP, and Ca^2+^ uptake was then determined as described in the Materials and Methods section. Results are expressed relative to control (no added NADP) as mean ± SEM, *n* = 3. Compared to control, statistical analysis was done using one‐way ANOVA with Dunnett's post hoc analysis (*P < 0.05; ***P < 0.001).

**Figure 3:**
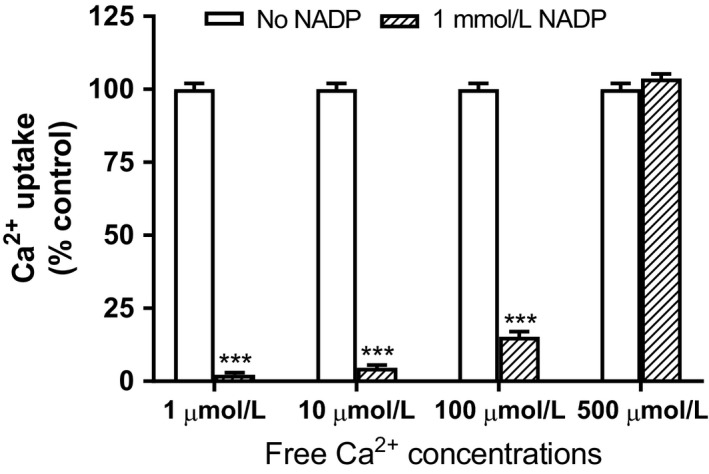
Effect of NADP and disparate free calcium concentrations on liver microsomal Ca^2+^ uptake. Isolated rat liver microsomes were incubated with 1 mmol/L NADP in buffer with disparate concentrations of free calcium. Calcium uptake was then determined as described in the Materials and Methods section. Results are expressed relative to control (no added NADP) as mean ± SEM, *n* = 3, ***P < 0.0001.

With NADP concentrations ranging from 0.01 to 2 mmol/L, there was a concentration‐dependent reduction in calcium uptake (Fig. 2). At 1 mmol/L NADP, calcium uptake was ~20% of control, and nearly completely quenched at 2 mmol/L. Moreover, the magnitude of the inhibitory action of NADP on calcium uptake was contingent on the free calcium concentration in the assay (Fig. 3). At a free calcium of 500 μmol/L, any inhibitory effect of 1 mmol/L NADP was indiscernible.

**Figure 4:**
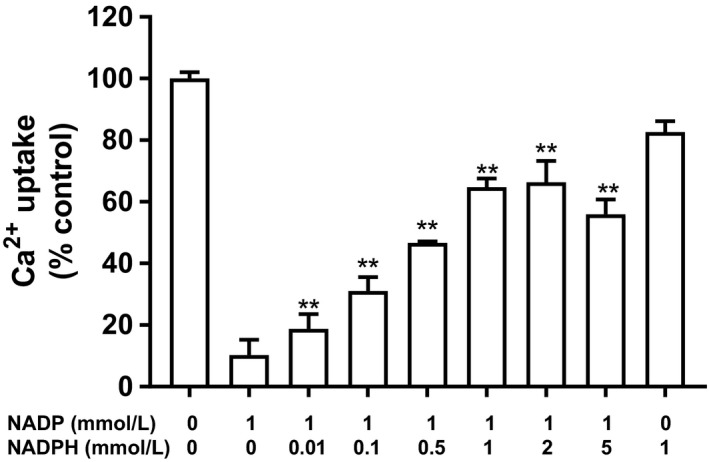
Effect of combination of NADP and NADPH on Ca^2+^ uptake in rat liver microsomes. Microsomes were incubated with 1 mmol/L NADP combined with 0.01–5 mmol/L NADPH for 5 min. Following which, Ca^2+^ uptake was then determined as described in the Materials and Methods. Results are expressed relative to NADP alone (1 mmol/L) as mean ± SEM, *n* = 3 (**P < 0.01).

**Figure 5:**
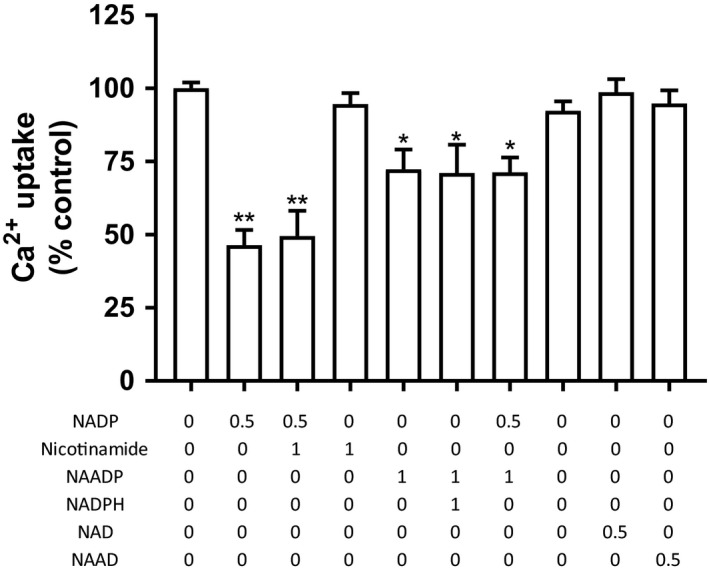
Effect of nicotinamide compounds, and combinations thereof, on Ca^2+^ uptake in rat liver microsomes. Beneath each bar, the compound(s) added to the uptake assay is listed (all concentrations are in mmol/L). Microsomes were incubated for 5 min with NADP, NAADP, NAD, NAAD, nicotinamide alone, or in combinations, calcium uptake was then determined as described in the Materials and Methods section. Results are expressed as mean ± SEM, *n* = 3. Statistical analysis was done using paired *t* test (*P < 0.05; **P < 0.01) versus control (no addition). NAADP: Nicotinic acid adenine dinucleotide phosphate, NAD: Nicotinamide adenine dinucleotide, NAAD: Nicotinic acid adenine dinucleotide.

Adding NADPH to the assay, thereby changing the pyridine redox state, significantly reversed the uptake inhibition by 1 mM NADP (Fig. 4) – however, even at 5 mmol/L NADPH, calcium uptake did not return to control values (no NADP). In Figure 5, various compounds containing a nicotinamide moiety were tested. With the exception of NAADP, no other nicotinamide compounds altered calcium uptake. Also, because high concentrates of NAADP desensitize its receptor, both high and low concentrations were also tested: at 1 μmol/L, there was no effect on calcium uptake, but at 1 mmol/L there was a 28% reduction (Galione 2011). Nicotinamide alone had no consequence.

**Figure 6:**
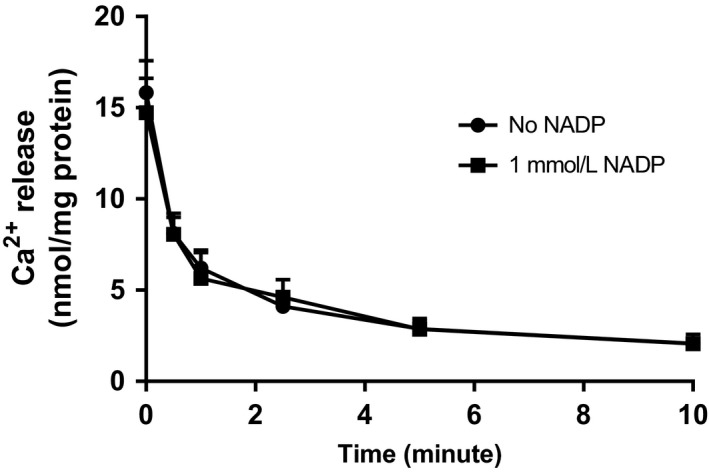
Effect of NADP on microsomal calcium release. Liver microsomes were preloaded with 1 mmol/L CaCl_2_ and 40 μCi/mL ^45^CaCl_2_, and the efflux was determined as described in the Materials and Methods section. Results are expressed relative to control (no added NADP) as mean ± SEM, *n* = 3. Statistical analysis was done using Student's *t* test (no differences were apparent).

When microsomes were preloaded with calcium, following which calcium was rapidly removed from the incubation media, there was a rapid outflow of the cation (Fig. 6). There was no difference in the loss of microsomal calcium in the presence/absence of NADP. However, insofar as the calcium uptake methodology requires considerably longer time duration (30 min), studies were also performed in which selective calcium efflux channels were blocked. This set of experiments was conducted to exclude the possibility that, as an alternative explanation, NADP was activating calcium release channels rather than directly stanching ER uptake. Specific channel inhibitors were incubated concomitantly with NADP during the assay (Ozawa 2010). Following channel blockage, the NADP effect on calcium uptake was then determined (Table 1).

**Table 1:** Effect of various calcium channel blockers, with and without NADP, on Ca^2+^ uptake in rat liver microsomes

Ca^2+^ channels	Inhibitors (10 μmol/L)	% control		
		Inhibitor alone	Inhibitor plus 0.5 mmol/L NADP	0.5 mmol/L NADP alone
NAADP	trans‐Ned‐19	90.7 ± 0.7	65.6 ± 0.8	61.8 ± 3.3
IP3	2APB	93.2 ± 1.8	44.2 ± 3.1	61.8 ± 3.3
RyR	RR	95.0 ± 2.2	43.9 ± 4.8	61.8 ± 3.3
	Dantrolene	95.7 ± 2.3	44.7 ± 4.7	61.8 ± 3.3

Microsomes were incubated for 5 min with trans‐Ned‐19, RR, 2APB or dantrolene at 10 μmol/L alone or in combination with 0.5 mmol/L NADP. Thereafter, calcium uptake was determined as described in the Materials and Methods section. Results are expressed as mean ± SEM. None of the channel inhibitors alone had a significant (*P* > 0.05) effect on uptake. NAADP, Nicotinic acid adenine dinucleotide phosphate; 2APB: 2‐Aminoethoxydiphenyl borate; RR, Ruthenium red.

Despite blocking ryanodine (RyR), inositol 1, 4, 5 – triphosphate (IP_3_R), and NAADP receptors with, respectively, ruthenium red and dantrolene, 2APB, and trans‐NED‐19, the NADP inhibiting effect on uptake was not attenuated (Mitchell et al. 2003; Galione and Ruas 2005). Unexpectedly, NADP inhibition of uptake in the presence of RyR and IP_3_ channel blockers was even more distinct. To sum, should NADP have opened an efflux channel, then inhibition of that conduit should have extinguished, or certainly reduced, the inhibitory effect of NADP on calcium uptake; however, with each channel inhibitor, this action was not observed.

**Figure 7:**
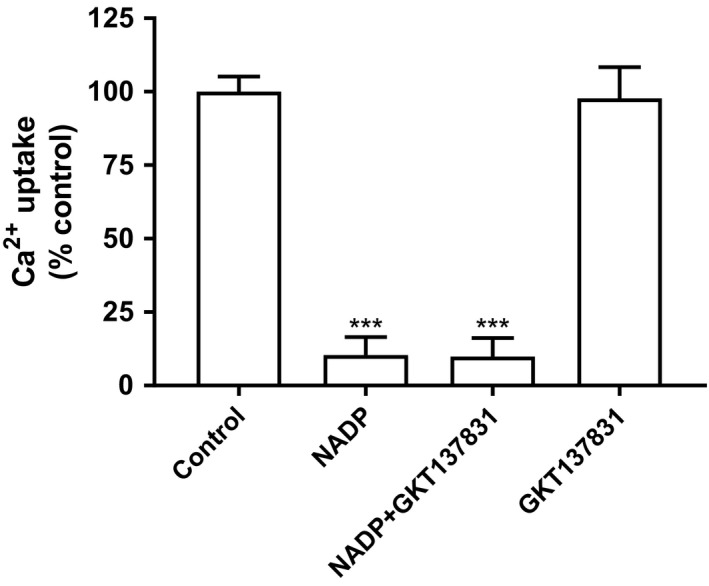
Effect of NADP and GKT137831 (a NOX4 inhibitor) alone, or in combination on Ca^2+^ uptake in rat liver microsomes. Microsomes were incubated for 5 min with 1 mmol/L NADP, 20 μmol/L GKT137831 or NADP combined with GKT137831, calcium uptake was then determined as described in the Materials and Methods. Results are expressed as mean ± SEM, *n* = 3, ***P < 0.001.

Both SERCA and RyR channels can be altered by NOX /NADPH oxidases, especially isoform NOX4 which is abundant in liver endoplasmic reticulum (Laurindo et al. 2014). Reactive oxygen species (ROS) generated by these oxidases are felt to be released in the ER lumen and, therefore, many ER calcium channels could be affected by oxidative/reductive processes (Gorlach et al. 2015). Thus, to explore the possibility that NADP was altering NADPH oxidase activity by adjusting the redox state, a potent NOX4 inhibitor (compound GKT13783) with an IC_50_ of 10.1 μmol/L was added to the uptake mixture (Fig. 7) (Altenhofer et al. 2015). No effect on the extent of NADP hindrance on uptake was detected.

**Figure 8:**
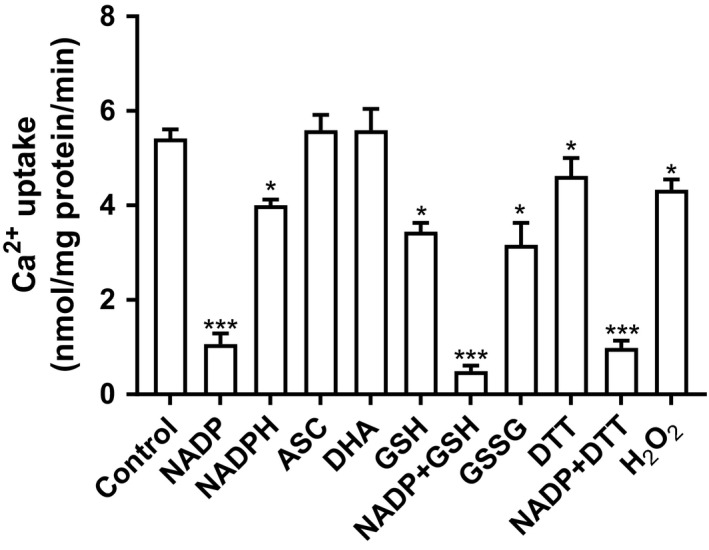
Effect of various oxidative and reductive compounds on Ca^2+^ uptake in rat liver microsomes. Microsomes were incubated with various modifiers as shown and the Ca^2+^ uptake during the 30 min assay was quantified. The concentration of each compound was 1 mmol/L. Results are expressed as mean ± SEM (*n* = 3). Statistical analysis was done using paired *t* test versus control (*P < 0.05; ***P < 0.001). N.S., not significant vs control; ASC, Ascorbic acid; DHA, Dehydroascorbic acid; GSSG, Glutathione disulfide; DTT, Dithiothreitol; GSH, reduced glutathione.

As aforesaid, ROS compounds can inhibit SERCA and ER calcium channels (Gorlach et al. 2015). In our microsomal assay, oxidizing compounds (H_2_O_2,_ glutathione disulfide) reduced calcium uptake, whereas dehydroascorbic acid did not (Fig. 8). As for the latter compound, its reduced form, ascorbate, likewise had no uptake effect. The mild inhibition by NADPH alone is unexplained but could be a direct effect or secondary to the generation of NADP via NADPH oxidases present on microsomes. Notably, however, the NADP restraint of uptake was not counteracted by the concomitant addition of reducing agents (DTT and GSH), inferring that ROS are not contributing to the NADP effect.

**Figure 9:**
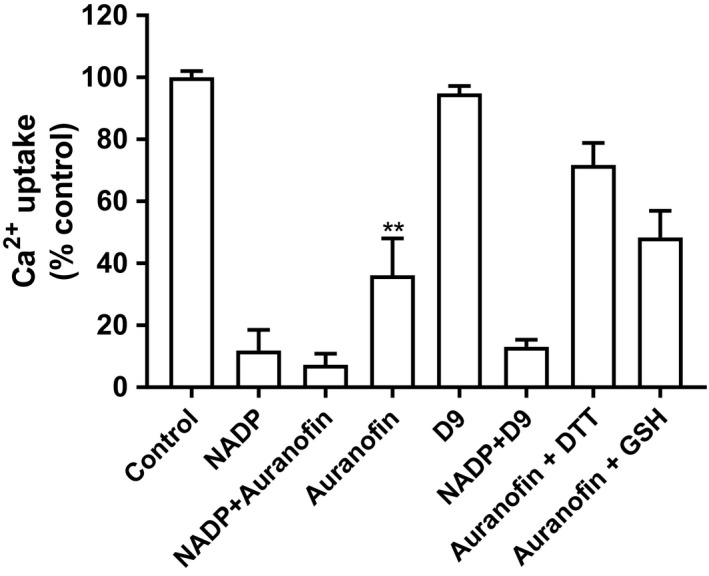
Effect of auranofin and D9 (both thioredoxin reductase inhibitors) alone, or in combination with NADP or reducing agents, on Ca^2+^ uptake in rat liver microsomes. Microsomes were incubated for 5 min with 1 mmol/L NADP, 20 μmol/L auranofin or D9 alone or NADP combined with auranofin or D9, calcium uptake was then determined as described in the Materials and Methods. Results are expressed as mean ± SEM (*n* = 3). Statistical analysis was done using paired *t* test (**P < 0.01 vs. control).

Insofar as NADP will alter the assay redox balance, a series of experiments were designed to address the possibility of a recently proposed ER‐embedded membrane oxidoreductase which is a pivotal component of the flavoprotein thioredoxin reductase 1 (TNR1) sequence (Poet et al. 2017; Ellgaard et al. 2018). Accordingly, a gold (I) compound which is an irreversible inhibitor of TNR1, namely auranofin, was tested (Fig. 9). This compound alone significantly reduced calcium uptake by nearly 70%, yet another potent, more specific, gold (I) inhibitor (called D9) was ineffective. Inasmuch as auranofin can generate ROS, the addition of reducing agents (DTT, GSH) mitigated its inhibition.

**Figure 10:**
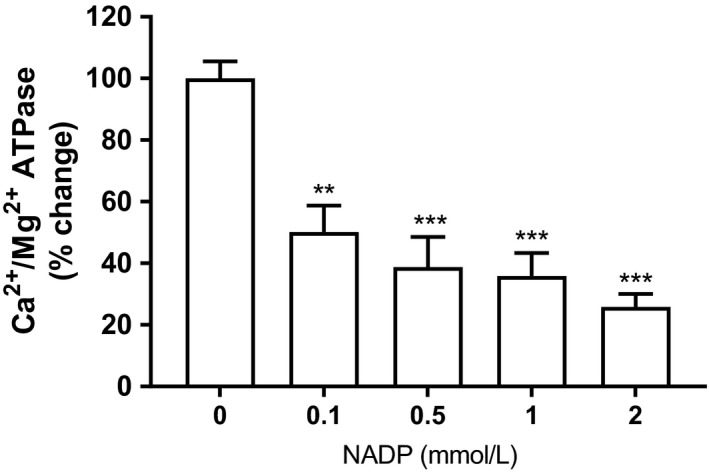
Effect of NADP on liver microsomal Ca^2+^/Mg^2+^‐ATPase activity. Isolated rat liver microsomes were incubated with the indicated concentrations of NADP, and Ca^2+^/Mg^2+^‐ATPase activity was then determined as described in the Materials and Methods section. Results are expressed relative to control (no added NADP) as mean ± SEM, *n* = 3. Statistical analysis was done using paired *t* test; *P* values are shown (**P < 0.01, ***P < 0.001).

Microsomal Ca^2+^‐ATPase activity, as determined by release of phosphate, was inhibited by NADP; however, the magnitude of which did not mirror the results observed in the uptake studies (Fig. 10). At 1 mmol/L NADP, there was a 64% reduction in ATPase activity, appreciably less than the corresponding inhibition on calcium uptake. However, only 14% of the total ATPase activity in liver microsomes is attributable to Ca^2+^‐ATPase, the bulk of the liberated phosphate is derived from Mg^2+^‐ATPase (i.e., thapsigargin insensitive) (de Moel et al. 1995).

## Discussion

The total pyridine nucleotide concentrations used in these experiments were within the physiologic ranges found in assorted tissues. Liver contains submillimolar total(free and bound) concentrations of pyridine nucleotides. For example, in hepatocytes, assuming approximately 2 mL of water per g dry weight, the total cellular concentration of NADP^+^ and NADPH in the cell is ~0.9 mmol/L (Tischler et al. 1977). As pertains to the corresponding nonphosphorylated nucleotides (NAD^+^ and NADH), the total cellular concentration is ~1.5 mmol/L. In liver, the cytosolic NADP concentrations are ~0.35 mmol/L based on 173 nmol/g wet weight and 53% of wet weight is intracellular water (Lindall and Lazarow 1964; Cieslar et al. 1998).

Both the cytosolic and ER redox states can modulate not only ER calcium exit channels, but also uptake via transporter calcium ATPases (e.g. SERCA2b) (Morris and Sulakhe 1997; Xu et al. 1997; Li and Camacho 2004; Zima et al. 2004; Gorlach et al. 2006; Ozawa 2010; Gorlach et al. 2015; Ushioda et al. 2016). Oxidation of protein sulfhydryl groups diminishes Ca^2+^‐ATPase activity (Horakova et al. 2013). Several pyridine containing compounds were tested, with or without the antioxidants GSH and DTT (Fig. 8). Not unexpectedly, oxidizing agents (H_2_O_2_ or GSSG) attenuated calcium uptake (Scherer and Deamer 1986; Morris and Sulakhe 1997; Xu et al. 1997; Hidalgo et al. 2005; Ozawa 2010; Gorlach et al. 2015; Ushioda et al. 2016). Yet the concomitant addition of reducing compounds (GSH or DTT) failed to prevent the NADP restriction of uptake‐ this infers that the nucleotide effect on uptake was extraneous to the generation of ROS, thereby inhibiting SERCA. In short, none of the inhibitors alone significantly inhibited or stimulated calcium uptake, nor did they change the NADP inhibition.

The uptake inhibition due to NADP was apparent even in the presence of specific inhibitors of calcium release channels, such as IP_3_ and RyR (but, arguably, liver ER membranes may be devoid of RyR) (Shoshan‐Barmatz 1990; Lilly and Gollan 1995; Mitchell et al. 2003; Hidalgo et al. 2004, 2005; Giunti et al. 2007; Saleem et al. 2014). This data infers that calcium influx is the site(s) of net uptake inhibition by NADP.

When NADPH was added to adjust the redox ratio, the NADP inhibition was blunted. The relevance of a redox ratio (NADH/NAD) was likewise demonstrated in permeabilized ventricular myocytes by assessing ER calcium egress via the RyR conduit (Zima et al. 2004). Our experiments corroborated the relevance of the pyridine nucleotide redox ratio affecting net ER calcium flux.

Another second messenger that mobilizes stored calcium, especially from acidic vesicles such as lysosomes, is nicotinic acid adenine dinucleotide phosphate (NAADP) (Galione 2011). This compound is a derivative of NADP, generated by ADP –ribozyme cyclase (CD 38), abundant in plasma membranes but also found, but to a lesser degree, in nonskeletal microsomes (Chini et al. 2002; Meszaros and Bak 1992). In the presence of nicotinic acid and low pH, the enzyme converts NADP to NAADP and nicotinamide‐in sum, a carboxyl moiety has replaced the NADP amide. Of note, in one relevant study, NADP had no effect on calcium release from sarcoplasmic reticulum (Hohenegger et al. 2002). There was a slight reduction (28%) in uptake at 1 mmol/L NAADP, but this did not modify the inhibitory action of NADP when the two compounds were combined. At 1 mmol/L concentration, NAADP reduced the net uptake by 28%; inhibition was not observed at 1 μmol/L. And although the inhibition by NADP is mitigated by NADPH, this was not the case with NAADP‐ namely, 1 mmol/L NADPH did not counter the 25–30% inhibition due to NAADP (Fig. 5). Nicotinamide (1 mmol/L) alone did not alter calcium uptake. As a final point, it should be noted that the inhibition by NADP was not secondary to the generation of NAADP. Although this latter nucleotide can be catalytically derived from NADP by a base‐exchange reaction via ADP‐ribosyl cyclase, in which nicotinic acid is the substrate, the presence of both (enzyme and substrate) in liver microsomal preparation is highly improbable.

Two previous studies concluded that NADPH>NADP slightly inhibited calcium uptake in hepatic microsomes (Prasad et al. 1986; Erickson et al. 1987). In these experiments, NADPH was not directly added to the assay, rather it was generated via the oxidation of G6P by the inclusion of G6P dehydrogenase. This would, in turn, lower the concentration of G6P in the assay, a phosphorylated compound which detains calcium in the ER lumen (possibly by liberating phosphate, forming insoluble salts to thwart backflow) (Wolf et al. 1986; Romani et al. 1988; Fulceri et al. 1990; van Schaftingen and Gerin 2002). Because the ER membrane is permeable to oxalate, this anion serves a calcium‐impounding role in nearly all microsomal uptake experiments. Nevertheless, in terms of net calcium sequestration in microsomes, the two compounds (oxalate/G6P) together in our uptake assay are additive (data not shown). Consequently, by consuming G6P to synthesize NADPH, this leads to an attenuation of calcium uptake, not by a direct inhibition of microsomal uptake by NADPH, but rather by decreasing the G6P capacity to sequester ER lumenal calcium. This can lead to a specious conclusion that NADPH per se inhibits calcium uptake.

Intriguingly, both reductive and oxidizing processes coexist in the ER (Margittai et al. 2015). Inasmuch as the ER lumen is predominantly an oxidized thio‐disulfide milieu, precisely how reducing electrons can coexist in this setting is unresolved. For example, a reduction process in the ER involves the conversion of cortisone (inactive) to cortisol (active) via the bidirectional enzyme.11‐β hydroxysteroid dehydrogenase. And to correct the noxious disulfide bonds of misfolded proteins in the ER, protein disulfide isomerases require electrons from elements in the cytosol (Poet et al. 2017; Ellgaard et al. 2018). Recent evidence suggests that these cytosolic electrons, derived ultimately from the nutrient G6P, are delivered to the ER lumen via cytosolic thioredoxin reductase 1 (TNR1) and NADPH (Dagnell et al. 2017; Poet et al. 2017; Ellgaard et al. 2018). To accomplish this task, reducing equivalents are ultimately shuttled across the ER membrane by a yet unidentified membrane‐embedded oxidoreductase (Poet et al. 2017). Therefore, experiments were designed to address the possibility that the cytosolic thioredoxin reductase 1 pathway, by changing the ER redox state, could impact ion fluxes. Admittedly, under the experimental conditions, the existence of aTNR1pathway is unlikely insofar as isolated microsomes are devoid of soluble cytosolic enzymes. Although auranofin per se inhibited calcium uptake, a more specific inhibitor of TNR1 (termed D9) was ineffectual (Saccoccia et al. 2014; Zhang et al. 2014). Hence, calcium uptake inhibition by NADP is not acting through a thioredoxin cascade, and the effect of auranofin in all likelihood is secondary to its capacious and potent inhibition of flavoprotein pyridine oxidoreductases or through ROS production (Vint et al. 1994; Saccoccia et al. 2014; Varghese and Busselberg 2014; Wang et al. 2017).The latter likelihood is supported by the observation that reducing agents (GSH and DTT) tempered the inhibition from auranofin (Fig. 8).

Besides the TNR1 system, there exist at least seven selenoproteins in the ER, several of which modulate calcium flux (Appenzeller‐Herzog and Simmen 2016; Addinsall et al. 2018). Auranofin, acting nonspecifically, conceivably modifies several of these selenium‐containing flavoprotein oxidoreductases (Saccoccia et al. 2014). We found a 64% inhibition of calcium uptake by auranofin. Nevertheless, auranofin, by binding irreversibly to selenol motifs in enzymes, is promiscuously nonspecific, and may directly interact to unfavorably reduce activity of an array of diverse non selenoenzymes (e.g. adenylyl cyclase), or through the production of ROS (Lazarevic et al. 1992; Vint et al. 1994; Wang et al. 2017).

Given that ROS can modify ER release channels, as well as SERCA, a change in the pyridine nucleotide redox ratio could, in turn, regulate NOX NADPH oxidases present in the ER (Laurindo et al. 2014). To ensure that ROS were not generated from NOX NADPH oxidases, in particular, the NOX 4 isoform associated with ER, specific enzyme inhibitors were added to the uptake studies (Altenhofer et al. 2015). Suppressing NOX activity did not modify the NADP effect on calcium uptake, nor did the concomitant addition of compounds which can curb ROS oxidation (Table 1).

Given the low free calcium concentrations in the uptake assay, and the dependence of the % NADP inhibition on free calcium, any physiochemical sequestering of calcium by binding to other compounds should be considered. To wit, a calcium‐pyridine nucleotide complex could lower the free calcium concentration in the assay, thereby reducing uptake, and resulting in a spurious conclusion. As it turns out, the association constant for NADPH is twofold greater than NADP; hence, NADPH would be expected to be speciously more “inhibitory” in our uptake assay if a reduction in free calcium was responsible (Burkhard 1982).

In conclusion, NADP inhibits calcium uptake in hepatic microsomes and the site of action ostensibly involves the ingress component of net calcium uptake (Kilfoil et al. 2013). Many classes of ion channels are modulated by pyridine nucleotides and, of note, even fat synthesis can be attenuated by NADP acting directly on fatty acid synthetase (Erickson et al. 1987; Stern and Smith 1987; Tipparaju et al. 2005; Tamsett et al. 2009). Seemingly, for ER calcium uptake inhibition, the phosphate moiety of the pyridine nucleotide is a requisite insofar as neither NAD, nicotinamide, nor NAAD were inhibitory in our assay (Agledal et al. 2010). Presently unknown is whether NADP inhibition involves a cofactor mass action effect on an embedded ER oxidoreductase or, alternatively, that this nucleotide binds to a regulatory site within an ingress channel.

**Figure 11:**
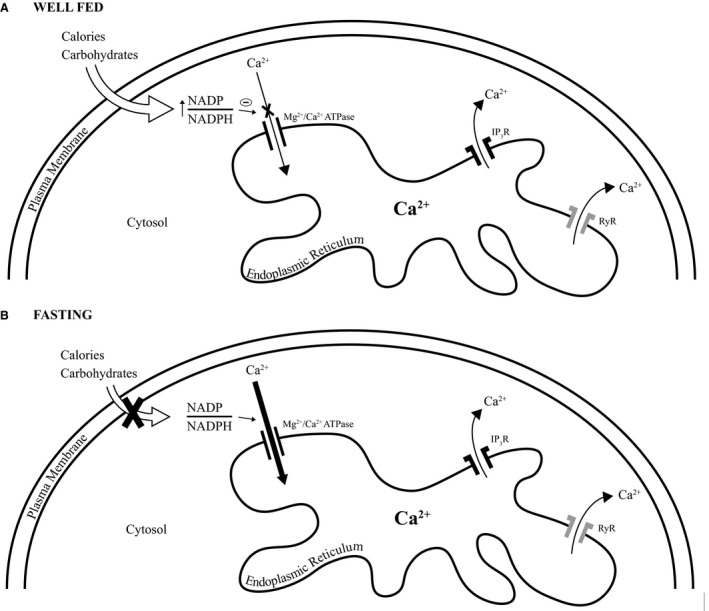
Schematic of the proposed nutritional regulation of pyridine nucleotide‐mediated endoplasmic reticulum calcium uptake. (A) In the well‐fed state, the cellular influx of carbohydrates and calories leads to an increase in the cytosolic NADP/NADPH ratio. This in turn inhibits endoplasmic reticulum calcium uptake. (B) In the fasting state, diminished cellular carbohydrate and calorie influx reduces the nutritional inhibition of calcium uptake. RyR, ryanodine receptor; IP_3_R, inositol 1, 4, 5 – triphosphate receptor

Lastly, as pertains to pathology, disturbed lipid and pyridine nucleotide metabolism, commonly encountered in obesity, impairs endoplasmic calcium uptake which, in so doing, begets ER stress (Luciani et al. 2009; Park et al. 2010; Fu et al. 2011). And, with obesity in mind, the amount of nutrient intake adjusts the intracellular redox state (Fig. 11). Case in point, in rodents that are well fed with a high sucrose diet, versus those that are fasting, the hepatic free (unbound) NADP/ NADPH ratio in the cytosol increases nearly fivefold to eightfold (Veech et al. 1969; Tischler et al. 1977; Laurindo et al. 2014). Hence, it is not inconceivable that the cytosolic accumulation of NADP, due to excess caloric intake, could have similar consequences on ER calcium uptake and stress as does disturbed lipid metabolism.

## Conflict of Interest

None declared.
